# Ophthalmology

**Published:** 1905-04-01

**Authors:** 


### OPHTHALMOLOGY.

**Foreign Bodies in the Eye, and their Removal with the Electro-magnet.**—Dr. Marple,[1] of New York, in an interesting paper on this subject, points out that particles, such as glass or copper, are very much more difficult to remove than those of steel or iron, the removal of which may be helped by the magnet. Fortunately, injuries with these particles do not occur as frequently, nor is their prolonged stay in the eye as liable to damage it as a visual organ. He quotes Haab as saying, with regard to such particles (not of steel or iron) it is often more advisable to leave them in the eye (unless, of course, they can be seen somewhere in the interior segment of the globe where they are accessible) than to open up the vitreous and probe around, as it were, in the dark with forceps and other instruments in search of them. To endeavour to remove such oftentimes damages the eye more than to leave it undisturbed. With regard to these steel and iron fragments, the introduction of the giant magnet, says Marple, as well as the improved technique in its use, has removed most of the disadvantages found previous to its invention. He sums up his paper as follows:—(1) An eye in which a piece of steel or iron is buried invariably deteriorates, and ultimately becomes blind (*siderosis bulbi*) if the foreign body is not removed, unless it becomes completely encapsulated. In many cases this degeneration is preceded by the symptoms of hemeralopia. (2) If the foreign body is in the anterior segment of the eye, the Haab magnet is almost universally used, at least to get the particle into the anterior chamber. (3) The injury in the great majority of cases, when it is in the anterior segment of the eye is not attended with a prolapse of the iris, and the occurrence of this complication makes it probable that the foreign body is not in the globe. This symptom, however, is not a reliable one in case the foreign body has made a large or irregular wound in the eye. (4) If the foreign body has penetrated into the vitreous, or posterior part of the globe, localisation either with the sideroscope, or $x$-rays, had better precede any attempt to extract it, especially if the lens is still transparent. After the particle has been localised it can be removed by way of the anterior chamber with the Haab magnet, or by opening directly into the sclera near where the particle has been located. As to which is the better method to be employed in this class of cases, is still a matter of discussion among opthalmologists. (5) If the symptom of pain cannot be elicited with the Haab magnet, this is to be interpreted as evidence (*a*) that there is no foreign body in the eye, (*b*) that it is enveloped, in recent cases, in a fibro-purulent exudation or a blood-clot, or in less recent cases that it is encapsulated, (*c*) that it has passed entirely through the globe, and is lodged partly or wholly in the orbital tissues (double perforation). A case by Berlin is quoted in relation to this last variety, where enucleation of the eye was already begun, when in cutting the tendon of one of the muscles the foreign body accidentally caught between the blades of the scissors at the back of the eye—it was seized with forceps and removed, the muscles already divided were reunited, and all irritation in the eye subsided.

**Radium Rays and their Action on the Normal and Blind Eye.**—Greef[2] reports the result of his official investigation of the optical properties of radium, particularly in respect to the somewhat sensational article entitled "Hope for the Blind" published by London of St. Petersburg. Greet says it is necessary to recognise that these rays are of two kinds. In the first place a species of fluorescence is excited in certain bodies, which then give off indirectly rays of ordinary light; and, secondly, the radium itself throws off peculiar visible emanations called radium rays. The true radium rays can be detected by holding the substance a short distance away from the eye after the retina has been habituated to the dark chamber. When the radium is at a distance of about 10 c.m. from the eye a peculiar, diffuse, sea-green radiance suddenly becomes visible, increasing as the eye is approached, and diminishing with greater distance from the substance. It is impossible to determine the direction of the rays, as they penetrate all structures and give the same effect if the radium is held at the side of the head as in front. It has been the view of some observers that this effect is the result of fluorescence induced in portions of the eye itself, which thus are excited to throw off ordinary light rays; this the author denies for several reasons, *e.g.* that the radium rays do not bleach the visual purple of the retina. London's experiments are misleading since he does not particularise exactly the visual condition of his patients. Once the functions of the rods and cones is destroyed, the eye becomes entirely unable to transmit visual concepts to the brain centres. London's patients who had a perception of light when brought before the radium screen, would have experienced the same sensation before a screen illuminated by ordinary means, while the radium rays proper cannot be used to give visual picture, since they cannot be refracted. At present there is therefore no prospect of help for the blind through the intervention of radium.

**Metastatic Gonorrhœal Ophthalmia.**—Dr. Sym,[3] in a paper on the above subject, draws attention to a conjunctivitis gonorrhœal in its origin, but differing from the ordinary gonorrhœal conjunctivitis caused by the direct transmission of the pus from the urethra to the conjunctiva. He says the variety of gonorrhœal conjunctivitis, to which I wish to draw attention, is entirely different; it has more in common with the joint affection, and with the iritis which not unfrequently follows gonorrhœa, it is not caused by direct infection, it affects both eyes at the same time; the condition might sometimes be called smart, but never violent; there is but little swelling of the lids, not at all an excessive degree of hyperæmia, but what there is is of the conjunctiva alone. The discharge, which is not copious, is rather thin, mucoid, perhaps, muco-purulent, and contains few or no gonococci. Ulceration of the cornea is rare and but insignificant when it does occur; indeed, it is not improbable that even this complication is due to some interloping germ. In the two cases mentioned by him the disease occurred in young men, in both several joints were attacked, and both yielded fairly easily to treatment—protargol and solution of hydrarg. perchloridi. We have lately come across a similar case—the patient was admitted to hospital as a case of ordinary rheumatism, but while in bed and taking salicylates for his rheumatism his right eye became inflamed. It was at first thought to be iritis, and this no doubt was due to the fact that he had several posterior synechiæ from an old attack of iritis; later we found there was really no iritis but merely hyperæmia, and that the condition was a conjunctivitis which yielded to warm solutions of boracic acid. The joint troubles cleared up and patient was discharged as cured. He returned, however, with a fresh joint attack and conjunctivitis in his left eye this time—in about 10 days—and for a week no treatment seemed to have any effect until he was put on potassium iodide when the rheumatism and conjunctivitis disappeared together after three days. Large doses of quinine are strongly advocated for this condition, and we were about to adopt this treatment if the iodide had failed—it is now two months since the attack was cured and we have every reason to believe there has been no recurrence, Sym concludes his paper by saying: "To my mind it is quite clear that there is a disease such as I have been describing; that it is rare, especially when one considers the frequency of gonorrhœa; and that it is a condition for which surgeons, and more especially those who are much consulted regarding cases of venereal disease, should be on the look out."

Mr. Percy Dunn,[4] in a post-graduate lecture on so-called sympathetic ophthalmia, finds fault with the obsolete nomenclature used in ophthalmic surgery. First he says: we often hear and read of the synonym "ciliary" blepharitis. Why "ciliary"? how is it possible to conceive of an inflammation of the lids without the follicles of the lashes being involved? Next the "crystalline" lens. Why "crystalline"? everyone in these days understands what is meant when reference is made to the lens of the human eye. To define it however, by the adjective "crystalline" is suggestive that another lens exists in the human body. Next we come to the expression "phthisis bulbi." What does this mean? The natural inference to be drawn from such a term would be that the eye was the seat of a general tuberculosis. As a matter of fact, however, this is not so. Its employment in the present day is for the purpose of making a fanciful distinction between eyeballs which have passed into an atrophic condition as the result of panophthalmitis, as compared with those in which the nutrition has failed owing to some disturbance of the integrity of the ciliary region. It is evidently considered that such a distinction is not fanciful but necessary, because while atrophy in the former case is not liable to be followed by harm to the fellow eye, on the other hand so-called sympathetic mischief may supervene when injury to the ciliary region is the cause of the atrophy. Again, what is meant by the word "strumous" as applied to certain ocular diseases? There is nothing strumous in ocular surgery any more than there is nowadays in general surgery or medicine. Thus it is scarcely needful to remark that the expressions "struma," "strumous," and "scrofulous" have become entirely obsolete, and now possess no scientific significance. Let me next proceed by referring to the large class of cases known under the name of "ophthalmia." Ophthalmia, says a writer in Quain's "Dictionary of Medicine," is a general term which might be used to express any morbid condition of the eye, but which is restricted by custom to the forms of inflammation which originate in the superficial structures of the organ, such as the varieties of conjunctivitis, or the phlyctenulæ which sometimes appear on the cornea, and give rise to shallow ulcers. With regard to the subject sympathetic ophthalmia, is it in strict accord with modern pathology to speak of an inflammation as sympathetic. What is the pathology of sympathy? I may refer to the modern views held with respect to the etiology of so-called sympathetic ophthalmia. (1) That the disease commences in the ciliary region of the injured eye and is caused by a specific micro-organism; and (2) that the micro-organism creates a toxin which is conveyed by continuity of tissue, presumably by the lymph channels to the ciliary region of the healthy eye, in which by its irritating presence it causes a severe form of irido-cyclitis. I submit, therefore, that the term "infective cyclitis" is more accurate for the purposes of nomenclature and is more in accord with the pathology of the disease than the two-fold inaccurate one of sympathetic ophthalmia.

[1] Med. Rec., June 1904. [2] Ibid., April 1904. [3] Edin. Med. Jour., Aug. 1905. [4] Lancet, Aug. 13, 1904.

